# Genomic clustering and co-regulation of transcriptional networks in the pathogenic fungus *Fusarium graminearum*

**DOI:** 10.1186/1752-0509-7-52

**Published:** 2013-06-27

**Authors:** Katherine Lawler, Kim Hammond-Kosack, Alvis Brazma, Richard MR Coulson

**Affiliations:** 1 European Bioinformatics Institute, Wellcome Trust Genome Campus, Cambridge CB10 1SD, UK; 2 Institute for Mathematical and Molecular Biomedicine, King’s College London, Hodgkin Building, London SE1 1UL, UK; 3 Department of Plant Biology and Crop Science, Rothamsted Research, Harpenden, Herts AL5 2JQ, UK; 4 Cambridge Institute for Medical Research, University of Cambridge, Cambridge CB2 0XY, UK

**Keywords:** Transcriptional networks, DNA-binding domains, mycotoxin biosynthesis, filamentous fungi, gene clusters

## Abstract

**Background:**

Genes for the production of a broad range of fungal secondary metabolites are frequently colinear. The prevalence of such gene clusters was systematically examined across the genome of the cereal pathogen *Fusarium graminearum*. The topological structure of transcriptional networks was also examined to investigate control mechanisms for mycotoxin biosynthesis and other processes.

**Results:**

The genes associated with transcriptional processes were identified, and the genomic location of transcription-associated proteins (TAPs) analyzed in conjunction with the locations of genes exhibiting similar expression patterns. Highly conserved TAPs reside in regions of chromosomes with very low or no recombination, contrasting with putative regulator genes. Co-expression group profiles were used to define positionally clustered genes and a number of members of these clusters encode proteins participating in secondary metabolism. Gene expression profiles suggest there is an abundance of condition-specific transcriptional regulation. Analysis of the promoter regions of co-expressed genes showed enrichment for conserved DNA-sequence motifs. Potential global transcription factors recognising these motifs contain distinct sets of DNA-binding domains (DBDs) from those present in local regulators.

**Conclusions:**

Proteins associated with basal transcriptional functions are encoded by genes enriched in regions of the genome with low recombination. Systematic searches revealed dispersed and compact clusters of co-expressed genes, often containing a transcription factor, and typically containing genes involved in biosynthetic pathways. Transcriptional networks exhibit a layered structure in which the position in the hierarchy of a regulator is closely linked to the DBD structural class.

## Background

Bacterial genes are organised into co-transcribed operons sharing a common promoter, with coding sequences present within operons frequently producing polypeptides of related function [1]. The genomes of nematodes and trypanosomes also exhibit polycistronic transcription of gene clusters [2,3], though most eukaryotic genes are generally considered to be monocistronic, each with its own promoter and transcription terminator [4]. This implies that eukaryotic genes do not have to be in close proximity to be co-expressed, and that their organization within a genome could be random. However, it appears that genes having similar and/or coordinated expression are often clustered [5]: the genes comprising the vertebrate β-globin cluster are organised according to the timing of their expression during development [6] with members of the mammalian homeobox transcriptional regulator loci arranged according to their spatial pattern of expression along developmental axes [7]. Additionally, in all crown-group eukaryotes (organisms found at the top of molecular phylogenetic trees) there is a significant tendency for genes from the same metabolic pathway to cluster [8]. This suggests that higher order genome organisation is linked to expression patterns.

Gene clusters have been described in fungi for phenotypes as varied as nutrient use, mating type and pathogenicity [9]. Further, genes for production of a broad range of secondary metabolites are located adjacent to one another; additionally a pathway-specific regulatory gene is often embedded within these clusters. The existence in budding yeast of a higher-order organization of genes across chromosomes is constrained by transcriptional regulation, with the target genes of most transcription factors positionally clustered within chromosomes [10]. The molecular mechanisms underpinning co-expression are not understood, though incidences seem to fall into two categories acting: (i) on a relatively local scale and dependant on *in cis* promoters in the immediate vicinity, and (ii) over broad genomic spans, possibly involving *in trans* chromatin state and positioning within the nucleus [5]. Price *et al.*[11] have hypothesized that as the amount of regulatory information required to specify an optimal expression pattern increases, evolving the optimal expression profile separately for each gene becomes more difficult, whilst creating an operon does not. Hence, co-expression in eukaryotes could reduce stochastic differences in gene expression and also synchronise fluctuations, or noise, in the levels of components of pathways and complexes [12].

The filamentous fungus *Fusarium graminearum* is a major cause of blight in cereal crops, resulting in heavy losses to grain yield and quality, which can be exacerbated by the contamination of grain with various mycotoxins that pose a serious threat to food and feed safety [13]. These secondary metabolites - not essential for survival – have been shown in a few cases to be synthesized by gene clusters. For example, the *TRI*-gene cluster contains up to 14 genes coding for proteins involved in production of harmful B-type trichothecene deoxynivalenol (DON) and its acetylated derivatives [14]. In *Saccharomyces cerevisiae*, clustering of essential genes increases the robustness of populations to mutation, and may provide a significant selective force shaping meiotic crossover distribution [15]. Little or no recombination is observed over long sections (megabases) across *F. graminearum* chromosomes, followed by much shorter regions displaying considerably higher than average recombination rates [16-18]. This contrasts strikingly with *S. cerevisiae* where high densities of crossing over are often present within just a few kilobases [19] compared with the several hundred kb in *Fusarium*. Here, the impact of this pattern of recombination in *F. graminearum* on the composition of co-expressed gene clusters was investigated, in conjunction with the genomic locations and protein-domain composition of the genes controlling the transcriptional process.

## Results

The *F. graminearum* genes encoding proteins associated with the transcriptional process were identified by protein family detection and profile matching (see Methods and Additional file 1: Table S1): of the 14,100 protein entries comprising the *F. graminearum* genome [17], 723 were linked to transcription (transcription-associated proteins, TAPs - Table 1). Sequences orthologous to these 723 TAPs were obtained from 56 complete eukaryotic genomes through sequence searching (‘Detection of *F. graminearum* TAP orthologues’ in Methods), and placed into one of five categories derived from the TAP reference set functional annotations, namely basal transcription factors and cofactors (B), RNA polymerase subunits (P), DNA binding (D), chromatin remodelling and histone modification factors (C) and others (O).

**Table 1:** **Distribution of *F. graminearum *genes homologous to the TAP reference set and matching DNA-binding domain HMMs**

**TAP class**	**Description**	**Count**	** Taxonomic distribution**			
			***Fgr***	***Pez.***	***Fungi***	***Euk***
B	Basal transcription factors and co-factors	63	4	10	15	34
C	Chromatin remodelling and histone modification	63	8	14	19	22
D	DNA-binding proteins	546	179	258	89	20
P	RNA polymerase subunits	27	4	4	4	15
O	Unclassified (CCR-NOT subunits, non-DNA-binding factors)	24	6	8	3	7
**Total**		**723**				

The TAP classes (B, C, D, P, O) were split into four taxonomic groups according to whether homologous genes were found only in *F. graminearum* (*Fgr*), only in *Pezizomycotina* (*Pez*) genomes, only in fungal genomes (*Fungi*), or widely detected within the eukaryotic genomes (*Euk*).

**Figure 1:**
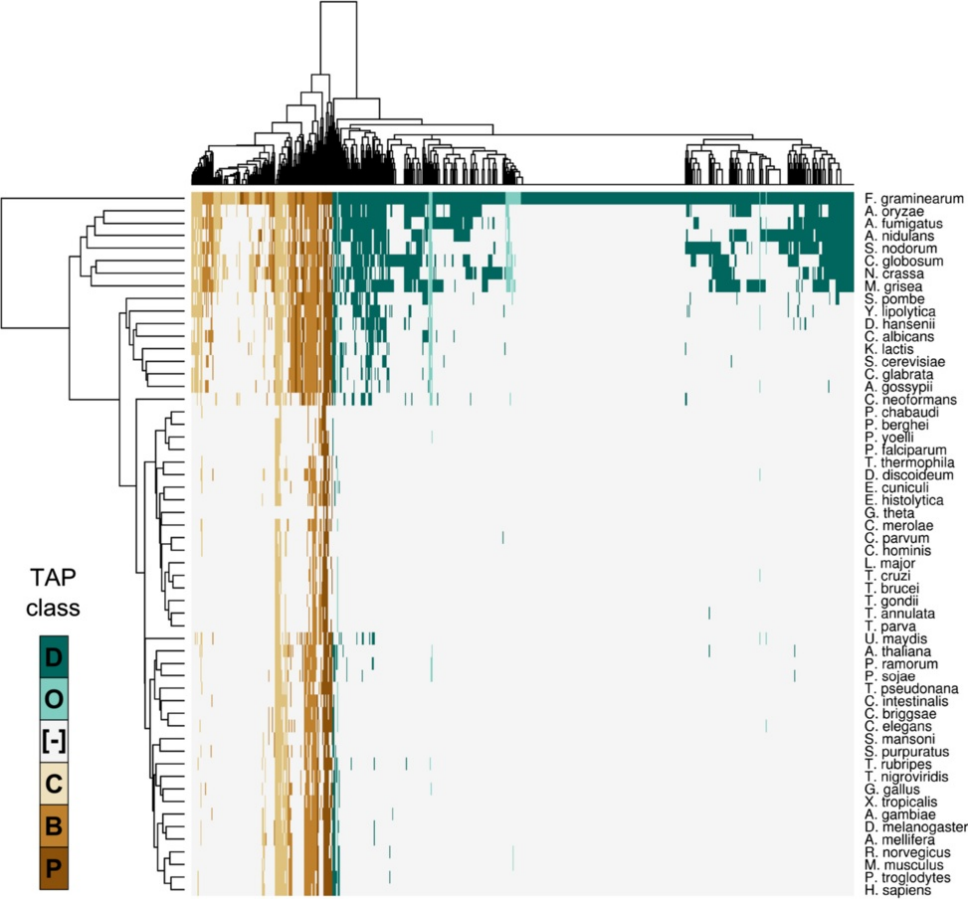
**Hierarchical clustering of the *F. graminearum *TAPs and their orthologues.** Rows represent genomes and columns homologous TAPs. A cell in the heatmap is filled if a homologue is detected and coloured according to the TAP class (D, O, C, B, P – defined in Table 1) of the matching *Fusarium* sequence. “[−]” indicates no homologue was detected.

The phylogenetic distribution of the TAP orthologues displays a high correspondence with their TAP class (Figure 1): one-third of the *F. graminearum* TAPs encoding DNA-binding proteins are only observed within the species (Table 1), and nearly a half (258/546) just have orthologues in filamentous fungi (*Pezizomycotina*). This contrasts with the four other categories, where nearly 60% of the B- and P-TAPs have orthologues in all the eukaryotic genomes analyzed.

### Chromosomal distribution of TAP functional classes

**Figure 2:**
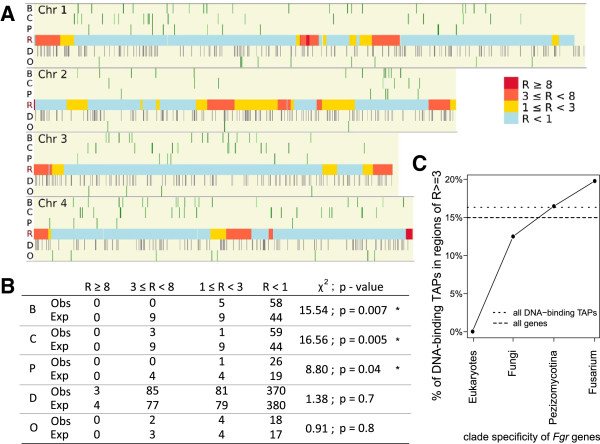
**Chromosomal distribution of *F. graminearum *TAPs. (A)** The genome was divided into four blocks representing high (R ≥ 8) to low (R < 1) recombination rate (cM/27kb). Chromosomal locations of the B, C, P TAPs and D, O TAPs are shown above and below, respectively, of the recombination map. **(B)** The observed (Obs) and expected (Exp) TAP gene counts are shown per recombination block for each TAP class, compared with a uniform distribution of TAPs over *F. graminearum* gene positions (χ^2^-test; *: *p* < 0.05). **(C)** Graph of the percentage of clade-specific DNA-binding TAPs lying in regions of high recombination (R ≥ 3). The percentage of all DNA-binding TAPs (short dotted line), and of all genes (long dotted line), lying in regions with high recombination rate are indicated.

The association between the degree of *F. graminearum* TAP conservation across eukaryotes and the recombination rate of the chromosomal region the TAP lies within was examined by delineating the genome into four groups by recombination rate (R, cM/27kb) [17,20]: these groups ranged in value from no or very low (R < 1) to very high (R ≥ 8) recombination rates. TAP classes B, C and P are under-represented amongst genes in regions of very high recombination rate, contrasting with their significant enrichment in areas of low or no recombination (Figure 2A,B). This under-representation is also seen with DNA-binding TAPs (D-TAPs) having homologues in metazoan genomes (Figure 1), as none are seen in areas of high or very high recombination (R ≥ 3). However, the percentage of D-TAPs increases in areas of high recombination as the clades become taxonomically less diverse i.e. twice the proportion of *Fusarium*-specific D-TAPs lie in these regions compared with those that have homologues in the fungal species examined (Figure 2C). These observations imply that proteins highly conserved, and associated with the transcriptional process, reside within areas of minimal recombination.

### Condition dependence of TAP gene expression

**Figure 3:**
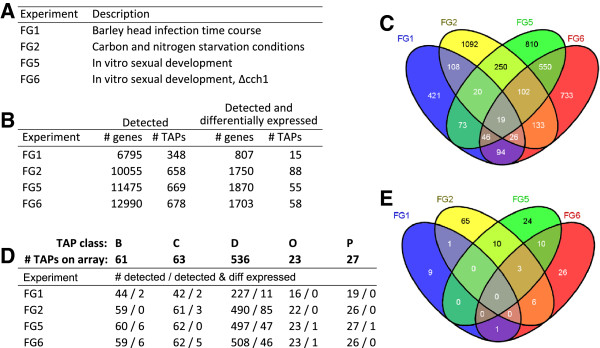
**Genes expressed in transcriptomics experiments. (A)** Gene expression data sets used in the study, listed by PlexDB identifier and description. **(B)** Number of genes expressed (Detected – ‘# genes’) and the number which are TAPs (‘# TAPs’ ) within each *F. graminearum* transcriptomics data set, and number that are also differentially expressed (‘Detected and differentially expressed’). **(C)** Venn diagram [21] summarizing the distribution of differentially expressed genes across the four sets of conditions. **(D)** Number of TAPs in each TAP class which were detected, or detected and differentially expressed, within each experiment (B, C, D, O, P; Table 1). **(E)** Venn diagram showing the overlap of DNA-binding TAPs differentially expressed in the microarray experiments.

An analysis of transcriptome data was undertaken to define TAP expression patterns and co-expressed gene clusters under a variety of environmental conditions. Several microarray gene expression data sets were selected: three spanning the *F. graminearum* lifecycle (FG1, infection of susceptible barley ears; FG5 & FG6, sexual spore development *in vitro*) and one a comparison of mycelium growth under nutrient rich and two different nutrient limiting conditions (FG2) (Figure 3A and Methods). Overall, 4,477 of the 13,830 genes represented on the array (32%) were identified as differentially expressed within at least one data set (Figures 3B and C, Additional file 1: Table S2), of which over two-thirds were observed to be differentially expressed only in one experiment (3,056 – the sum of the unique portions of the four gene sets, Figure 3C). Similarly, one-third of the DNA-binding TAPs - 155 (the union of the four DNA-binding gene sets) of the 536 represented on the array - were found to be differentially expressed (Figure 3D, E), again with the majority (80%) in only one experiment (Figure 3E). These analyses suggest there is an abundance of condition-specific control of *Fusarium* transcript levels. Within each of the four experiments, on average 15% of expressed non-TAPs, compared with 8.6% of expressed TAPs show differential expression; additionally, of all the differentially expressed DNA-binding TAPs, none exhibit altered expression levels in all four experiments i.e. the intersection of all four sets of these genes is zero (Figure 3E).

**Figure 4:**
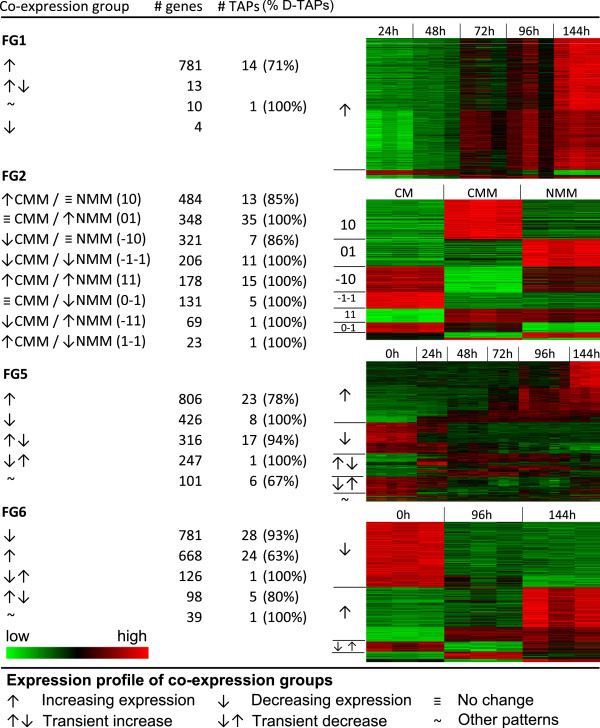
**Co-expression groups derived from transcriptomics experiments.** Column one shows the expression pattern observed in each co-expression group for a given data set, and columns two and three show the number of genes (‘# genes’) and TAPs (‘# TAPs’), respectively, with this expression profile. ‘% D-TAPs’ indicates the percentage of DNA-binding TAPs in the co-expression group. The final column shows the heatmaps corresponding to the various co-expression groups; increasing (red) and decreasing (green) expression is shown relative to the initial time point for the developmental and infection studies, and relative to complete media for the nutrient-limited conditions.

Gene expression patterns for probesets differentially expressed during the time course experiments were merged on the basis of similar temporal behaviours to create co-expression groups. This procedure compared subsequent timepoints with the earliest timepoint, to ascertain whether the expression level of the probeset was either stably (↑ or ↓, Figure 4) or transiently (↑↓ or ↓↑, Figure 4) altered. For example in FG5, 806 and 426 display stable increased (↑) and decreased (↓) levels, respectively, with 316 and 247 exhibiting transient elevated (↑↓) and reduced (↓↑) levels (Figure 4). All other changes in behaviour are indicated by a tilde (~). In the co-expression groups observed in the FG1, FG5 and FG6 microarray experiments, stable alterations in transcript levels predominate over transient ones.

### Genomic clustering of co-expressed genes and TAPs

**Figure 5:**
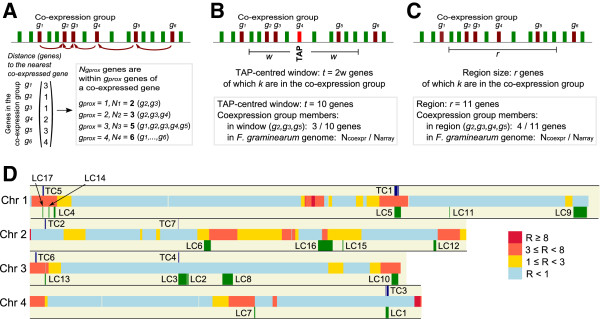
**Methods testing for chromosomal clustering of co-expressed genes. (A)** Genomic clustering of co-expressed genes. Within a given co-expression group (dark red), for each gene the distance to the nearest member was recorded. The total number (*N*_*gprox*_) of genes lying within *g*_*prox*_ genes of a co-expressed gene was counted and compared to uniform sampling from the genome. **(B)** TAP-centred clusters of co-expressed genes. For each TAP (red), the genes lying within a window spanning *t* adjacent genes around the TAP were tested for enrichment for co-expressed genes (dark red). The window size *t* was increased incrementally (*t* = 1 to *t* = 20). **(C)** Localized clustering of co-expressed genes. Each genomic region was tested for enrichment of co-expressed genes using PGE. **(D)** Chromosomal locations of the seven TAP-centred (TC) and seventeen localized (LC) gene clusters detected by the TAP-window and PGE methods, respectively.

Target genes of transcription factors are positionally clustered within budding yeast chromosomes [10], and in filamentous fungi there are secondary metabolite gene clusters known to be co-regulated (e.g. aflatoxin/sterigmatocystin and trichothecene biosynthesis in *Aspergillus* and *Fusarium*, respectively [22,23], and reviewed in [9,24]). To examine whether the genes present in the co-expression groups defined from the *Fusarium* transcriptomics experiments (Figure 4) also exhibit close proximity within the genome, several clustering methods were employed (Figure 5).

Global tests of clustering of co-expressed genes amongst all genes on the genome would not reveal densely packed, localized clusters in a background of sparsely distributed co-expressed genes, i.e. they would not necessarily distinguish localised organisation in a noisy background. To look for initial evidence of such clusters, the distance (in gene number) between consecutive pairs of co-expressed genes was measured within each of the 22 co-expression groups (Figure 5A; see Methods). In 9 of the 22 co-expression groups, significantly more genes were observed to be in close proximity (up to 10 genes apart on the genome) than expected if they were randomly distributed (Additional file 2: Table S3). This is consistent with the existence of localized clusters, but does not distinguish between tightly packed co-expressed clusters or dispersed proximal pairs of co-expressed genes.

**Figure 6:**
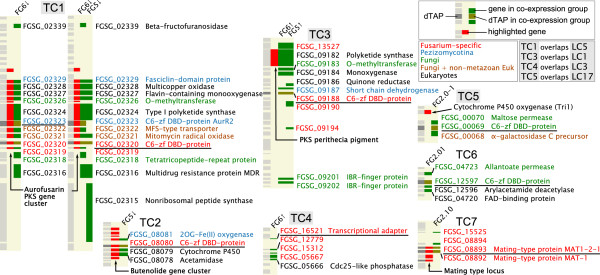
**TAP-centred clusters. The eight TAPs (underlined) located within the seven regions significantly enriched for co-expressed genes are shown.** Each cluster is displayed as three columns with the left showing the genes on the chromosome, the middle displaying genes of interest (red), and the right the members of the TC (non-DNA-binding TAPs shown in green, with D-TAPs in brown). The colour of the FG gene IDs (next to the chromosomal locations of the TC members) indicates taxonomic specificity, i.e. in which phylogenetic groups homologues are found. Alongside this identifier is given a functional annotation derived from the sequence searches and protein family detection. Abbreviations: C6-zf DBD-protein, Zn(II)_2_Cys_6_ DNA-binding protein.

The manner of localized clustering in gene order within the co-expression groups was further investigated by examining the expression patterns of genes in the vicinity of each differentially expressed TAP (Figure 3). For each differentially expressed TAP, the number of genes within the same co-expression group was counted for a variable window size of 2 to 40, i.e. 1 to 20 genes on each side of the TAP (Figure 5B). Fisher’s exact test was used to determine significance of enrichment of group members for a given window size, and resulted in the detection of seven TAP-centred clusters (TC; Figures 5D and 6). Five of the TAP-centred clusters (TCs) were observed in co-expression groups exhibiting non-random genomic distributions (Figure 5A). TC3 contains a gene encoding a polyketide synthase (PKS3/PGL1 – an enzyme involved in synthesizing black perithecium pigment) and it has been suggested that the sequences surrounding this PKS gene form a co-regulated group [18,25,26]. Furthermore, the members of TC3 show increasing expression during progression from vegetative mycelia to mature perithecia (FG5, FG6) reflecting an elevation in pigment synthesis during sexual development. Three out of the five genes present in the *F. graminearum* mating-type locus [27,28] were shown to comprise a co-expressed cluster (TC7), with this cluster containing an additional adjacent gene. Members of TC5 exhibit decreased levels of expression in nitrogen-minimal conditions and are highly homologous to the budding yeast MAL1 locus [29] which also contains a maltase, a Zn_2_(II)Cys_6_ DNA-binding factor and a maltose permease.

**Figure 7:**
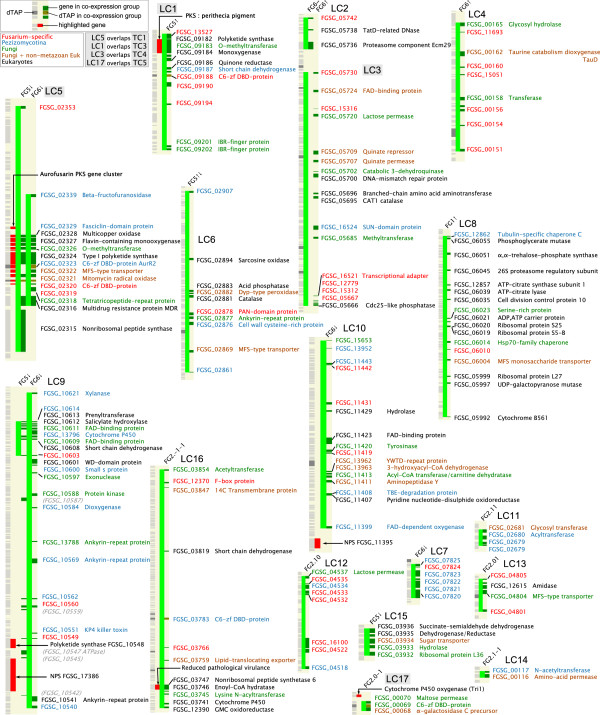
**Localized clusters identified by PGE.** The FgraMap images for the LCs have an additional column (solid light green) indicating the extent of the LC regions. A key in the upper left hand corner indicates which LC and TC clusters have the same chromosomal locations.

The presence of localized clusters (LCs) independent of the presence of a proximal co-expressed TAP was investigated using the Positional Gene Enrichment (PGE) tool [30] (Figure 5; see Methods). Seventeen LCs were identified as significantly enriched for co-expressed genes (Figures 5 and 7; LC1-17). Four were identified as TAP-containing (Figures 6 and 7) as described above. Two further classes of cluster were observed: ones where there were no intervening genes that were not members of the same co-expression group (LCs 7, 11, 13, 14, 15 and 17), and having an average size of five genes. The other cluster type showed much less compactness with an average size of 64 genes, of which around a third were members of the co-expression cluster. Several of these clusters contain sequences encoding nonribosomal peptide synthetases (NPS) – enzymes producing a wide range of mycotoxins and linked to *Fusarium* pathogenicity [31,32].

Putative protein functions for unannotated, co-expressed genes lying in TCs and LCs were derived from sequence and protein domain homology, and linking the results of these searches to Gene Ontology entries (Methods and Additional file 1: Table S5). Most of the 170 non-TAP genes appear to play a role in secondary metabolism (Figures 6 and 7), with 42% and 22% associated with metabolic and biosynthetic processes, respectively (Table 2). Hence, most of the co-expressed genes exhibiting positional clustering appear to encode proteins associated with secondary metabolism, indicating that co-regulation of gene clusters is primarily associated with controlling the biosynthesis of mycotoxins or other metabolites.

**Table 2:** **GO Slim assignments of the 170 non-TAP differentially expressed genes which are present in TCs and LCs (Figures **6**and**7**)**

**GO Slim term**	**% of TC/LC genes**	**GO Slim ID**
Metabolic process	42%	GO:0008152
Cellular process	39%	GO:0009987
Biosynthetic process	22%	GO:0009058
Macromolecular metabolic process	16%	GO:0043170
Regulation of biological process	16%	GO:0050789
Transport	14%	GO:0006810
Response to stimulus	12%	GO:0050896
Multicellular organismal development	11%	GO:0007275
Cell communication	10%	GO:0007154
Catabolic process	9%	GO:0009056
Nucleobase, nucleoside, nucleotide and nucleic acid metabolic process	8%	GO:0006139
Cellular amino acid and derivative metabolic process	6%	GO:0006519
Cell death	2%	GO:0008219
Cell differentiation	2%	GO:0030154
Behaviour	1%	GO:0007610
Pathogenesis	1%	GO:0009405

The percentage of these genes annotated with each term is shown.

### Defining global transcriptional regulators

Co-regulation of *Pezizomycotina* gene clusters encoding components of secondary metabolism pathways is partly coordinated through ‘narrow’- and ‘broad’-domain transcription factors [9], proteins primarily containing either Zn(II)_2_Cys_6_ or Cys_2_His_2_ amino acid motifs, respectively. In FG1 there is an enrichment of genes expressed that code for proteins containing bZIP, GATA and Cys_2_His_2_ DNA-binding domains (DBDs): significantly more Cys_2_His_2_-containing genes are expressed than would be expected given the overall proportion of DNA-binding TAPs expressed (Fisher’s exact test, p<0.0005; 55 of the 93 Cys_2_His_2_-D-TAPs are detected compared with 227 D-TAPs out of the 536 on the microarray (Figure 3D)). Conversely, a significant depletion in the expression of ‘narrow’-domain Zn(II)_2_Cys_6_ transcription factors is observed (Fisher’s exact test, p<10^-6^; 105 out of the 325 represented on the microarray).

The identification of genes controlling transcription of the members of co-expression groups was facilitated by Kumar *et al.*[33]: they reported the presence of 326 DNA-motifs located in upstream promoter regions of *F. graminearum* genes and conserved in *Fusarium* genomes. The enrichment of these DNA-motifs was tested in a region 600 bp upstream of the transcriptional start site of the genes in each co-expression group. Of the 326 motifs, 113 were enriched in at least one co-expression group (Additional file 1: Table S6). These enriched motifs could act as binding sites for global transcriptional regulators, i.e. transcription factors controlling the expression levels of a significant proportion of co-expressed genes (see Methods). To classify the DNA-binding domains present within these putative transcriptional regulators, *S. cerevisiae* motifs were searched and significant matches determined. *F. graminearum* proteins with significant homology to the associated budding yeast DNA-binding proteins were considered as global regulators (Additional file 2: Figure S2).

**Figure 8:**
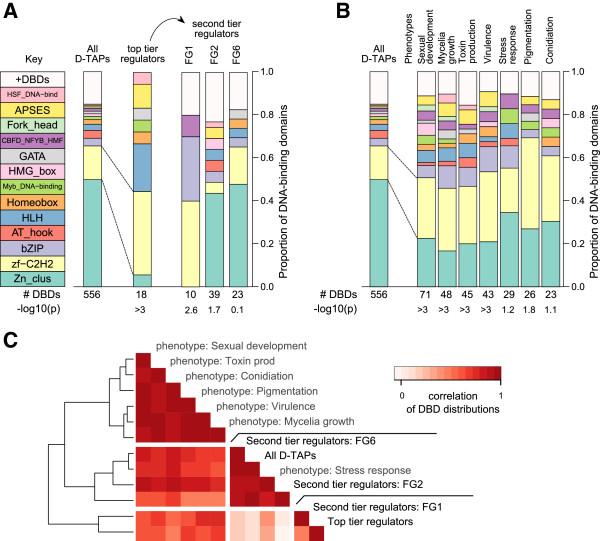
**Distribution of DNA-binding domains among *F. graminearum *transcriptional regulators. (A)** Barplots showing the distribution of DNA-binding domains present in D-TAPs (‘All D-TAPs’), and top- and second-tier regulators. ‘#DBDs’: number of DNA-binding domains detected; p-value (displayed as ‘–log10(p)’) obtained from Fisher’s exact test when comparing the top and second-tier distributions with the background. **(B)** Distributions of the pTF DBDs. **(C)** Heatmap visualizing the Pearson correlation coefficients of the DBD abundances – the pairwise correlation between the fraction of background, top and second-tier regulator and pTF DBDs.

Putative *F. graminearum* DNA-binding proteins were assigned to 31 enriched motifs (Additional file 2: Table S7): two-thirds of these 18 sequences contain either Cys_2_His_2_ or helix-loop-helix domains (Table 3). This deviation in the distribution of DNA-binding domains (DBDs) amongst the global regulators from the background distribution of DBDs was found to be highly significant (Figure 8A; p < 0.001, Fisher’s exact test). D-TAP-encoding genes present in co-expression groups and containing motifs potentially bound by global regulators in the upstream promoter region (‘second-tier regulators’) show different DBD-distributions from these top-tier (global) regulators. These second-tier regulators identified in FG2 and FG6 have similar DBD-distributions to all D-TAPs in the genome. In contrast, those in the FG1 infection experiment predominantly contain Cys_2_His_2_ and bZIP domains.

**Table 3:** ***F. graminearum *global regulators determined by homology to *S. cerevisiae *DNA-binding motifs**

***F. gr. *gene ID**	**DBD**	**# Enriched co-expression groups**	**Enriched co-expression groups**
FGSG_04220	APSES	1	FG2.11
FGSG_10384	APSES	1	FG2.11
FGSG_08634	GATA	1	FG2.01
FGSG_00750	HLH	2	FG1↑, FG2.11
FGSG_01307	HLH	2	FG1↑, FG6↓↑
FGSG_02814	HLH	4	FG1↑, FG2.11, FG6↓, FG6↓↑
FGSG_05567	HLH	4	FG1↑, FG2.11, FG6↓, FG6↓↑
FGSG_09043	Homeobox/zf-C2H2	1	FG2.11
FGSG_06359	HSF_DNA-bind	1	FG6↓
FGSG_13911	Myb_DNA-binding	1	FG2.01
FGSG_01298	zf-C2H2	1	FG6↓
FGSG_01341	zf-C2H2	2	FG1↑, FG2.10
FGSG_01350	zf-C2H2	2	FG1↑, FG2.10
FGSG_02743	zf-C2H2	2	FG1↑, FG2.10
FGSG_06311	zf-C2H2	1	FG6↓
FGSG_06871	zf-C2H2	4	FG1↑, FG2.-10, FG2.-11, FG6↓
FGSG_12970	zf-C2H2	1	FG6↓
FGSG_08010	Zn_clus	2	FG1↑, FG2.10

The gene identifier of the putative regulator (‘*F. gr.* gene ID’) along with its DBD-composition (‘DBD’), and the number and details of co-expression groups enriched for an associated DNA-motif are shown.

Son *et al.*[34] performed a systematic analysis of seven phenotypes of mutants in *F. graminearum* transcription factors. The DBDs present in these transcription factors was obtained from the HMM searches of the *F. graminearum* genome (see Methods). Comparison of the DBD-distributions with the background revealed enrichment of Cys_2_His_2_ and a depletion of Zn(II)_2_Cys_6_ domains (Figure 8B): 40% of the top-tier regulators contain Cys_2_His_2_ domains, with only 6% containing Zn(II)_2_Cys_6_ domains – an order of magnitude reduction compared with the background. This pattern of increased abundance in Cys_2_His_2_-containing proteins is also observed with the phenotype-associated transcription factors (pTFs), with an average of 30% containing this DBD contrasting with a background level of 16%. Pairwise similarities in the DBDs present in global, second-tier and pTFs were visualized by hierarchical clustering of these correlations (Figure 8C). These analyses showed that FG2 and FG6 second-tier regulators, along with those regulating stress responses, are highly similar to the background distribution. The FG1 second-tier regulators and the global regulators appear to cluster together due to the high and low levels of TAPs containing Cys_2_His_2_ and Zn(II)_2_Cys_6_ domains, respectively. The DBD patterns amongst the pTFs exhibit a less pronounced deviation from the background but seem to form a distinct cluster of six phenotype groups with a more diverse range of DBDs.

## Discussion

Gene function and phylogenetic conservation were found to be related constraints on gene positioning at the whole genome level in *Fusarium*. Rates of recombination were associated with levels of protein sequence conservation: conserved TAP categories - basal transcription factors and cofactors, RNA polymerase subunits, and chromatin remodelling/histone modification factors - are predominantly found in regions of very low or no recombination, possibly reflecting their fundamental role in the transcriptional process. However, highly diverged DNA-binding proteins (potentially with regulatory roles) are more often present in regions of high recombination. This organisation of transcription factors may increase the rate of adaptive evolution in *Fusarium* by more readily allowing the formation of transcriptional networks with superior adaptation to the habitat of the fungus.

Genome-wide searches for positionally-clustered genes revealed several categories: compact groups, with some containing putative transcriptional regulators, and more dispersed groups with co-expressed members lying amongst non-coexpressed genes. This pattern of clustering is consistent with that described in other eukaryotic genomes and indicates a diversity of mechanisms for co-regulation [5]. The aurofusarin gene cluster was detected (TC1, Figure 6) around the TAP *AurR1/GIP2* which encodes a regulator required for cluster transcription [25,35,36], and the butenolide gene cluster containing a cytochrome P450 (TC2) around a gene encoding a Zn(II)_2_Cys_6_ zinc-finger protein thought to regulate this cluster [37]. Deoxynivalenol (DON) mycotoxin production only occurs during the infection of barley ears, and six of the fourteen *TRI* genes are upregulated in FG1: four lie within the *TRI8*-*TRI14* gene cluster [38] and two are disparate *TRI* genes [39], precluding their identification as a single gene cluster. The remaining members of this biosynthetic pathway do not exhibit significant differences in transcript levels, possibly reflecting the role of post-transcriptional mechanisms in controlling DON synthesis. Furthermore, Reyes-Dominguez *et al.*[40] show that chromatin modification plays a significant role in the regulation of the tightly-linked genes involved with mycelium pigment (aurofusarin) and DON biosynthesis. Together, these observations imply that *Fusarium* gene clusters are subject to multiple levels of co-ordinated regulation.

A hallmark of secondary metabolism genes – in contrast to genes involved in primary metabolism – is that they are clustered in fungal genomes [41]. Systematic annotation of genes within the TAP-centred and localized clusters (Figure 5D) shows a number with homology to polyketide synthases, non-ribosomal peptide synthetases and other types of enzymes that synthesize mycotoxins. Present in the TAP-centred cluster containing the polyketide synthase gene *PKS9* (TC3) is a sequence encoding a Zn(II)_2_Cys_6_ zinc-finger protein; this local transcription factor controls the production of novel fusarielins [42]. TC6 is a putative, novel co-regulated gene cluster, and its components could comprise a biosynthetic cluster: a transport protein (allantoate permease), a Zn(II)_2_Cys_6_ DNA-binding factor (transcriptional regulator), and putative modifying enzymes (a FAD-binding protein and a deacetylase). The majority of localised clusters have members with homologues either in *Fusarium* or other filamentous fungi only, and is suggestive of biosynthetic pathways producing *Fusarium*-specific mycotoxins. The close proximity of the genes encoding both enzymatic and regulatory functions, and comprising these positional clusters, may provide an evolutionary mechanism that facilitates adaption to a wide variety of environments.

The majority of differentially expressed TAP genes encode DNA-binding proteins (D-TAPs), consistent with a role of such sequences in controlling developmental programs and responses to environmental fluctuations. D-TAP differential expression was found to be predominantly condition-specific (Figure 3), suggesting that different sets of transcription factors orchestrate various regulatory events. Interestingly, on average within each transcriptomics experiment, an order of magnitude more non-TAPs than TAPs exhibited differential expression (Figure 3B), and consistent with the frequency of motifs identified in the promoter regions of functionally related genes [33]. This suggests that individual TAPs may control the expression of multiple genes.

A comparison with two yeasts showed conservation of transcription factors, their binding sites and the target genes regulated by these factors with *Fusarium* pathways known to respond to stress conditions or phosphate metabolism [33]. These observations were extended to identify the types of DNA-binding domains (DBDs) present in the putative transcriptional regulators defined from the co-expression groups. Most global regulators contain either Cys_2_His_2_ or HLH domains and may control expression across a number of conditions. Additionally, a Cys_2_His_2_ zinc-finger protein encoded within the trichothecene gene cluster (*TRI6*) has been shown to act as a global transcriptional regulator [43]. This increase of Cys_2_His_2_ and depletion of Zn(II)_2_Cys_6_ domains is also seen with transcription factors which when individually deleted produce mutant strains with a variety of phenotypes; however, the classes of DBDs present in these proteins are more complex, possibly reflecting greater diversity in the biological processes studied.

Two distinct patterns of DBDs are observed within the second-tier regulators. The barley ear infection (FG1) secondary regulators are enriched for transcription factors containing a bZIP domain, and their classes are similar in distribution to those of the top-tier regulators. This may indicate more elaborate transcriptional networks are employed during host infection as the bZIP containing transcription factor *ZEB2* can act as a local regulator: the zearalenone biosynthesis gene cluster consists of four members, three of which are regulated transcriptionally by the fourth - *ZEB2*[13]. The secondary regulators identified in nutrient-deprived conditions (FG2) and differentiation from mycelia to perithecia (FG6) contain Zn(II)_2_Cys_6_ domains predominately. Their DBD class distributions are highly correlated with those of the transcription factors linked to the stress response and the background distribution of D-TAPs; this suggests they may regulate directly the transcription of genes participating in the response to various stimuli and sexual development.

McCord and Bulyk [44] observed in yeast that bZIP, Cys_2_His_2_ and HLH-containing global regulators are enriched in regulatory hubs, contrasting with local Zn(II)_2_Cys_6_-containing transcription factors that are depleted, and implying this global/local nature of a regulator is a feature of its structural class. Hence, top-tier regulators could contain DBDs (e.g. Cys_2_His_2_ or HLH structures) able to bind more degenerate DNA sequences and so control the transcription of many genes, whereas the Zn(II)_2_Cys_6_ domain may only recognize highly-specific DNA binding sites ensuring restricted regulation of a gene cluster. This use of different classes of DNA-binding proteins at certain levels within a transcriptional network, could thus allow the evolutionary diversification of mycotoxin production through the gain or loss of sequences from biosynthesis gene clusters [17,18]. These resultant phylogenetic distributions may provide further insights into the role, organization and regulation of gene clusters in *Fusarium* and other emerging fungal threats [45].

## Conclusions

Proteins associated with the basal functions of transcription e.g. RNA synthesis, are encoded by genes lying in areas of the *Fusarium* genome with little or no recombination, contrasting with those performing roles in controlling gene activation. Systematic searches for gene clusters revealed compact groups usually containing DNA-binding proteins and more dispersed types; however, both seem to contain an abundance of genes whose products could partake in pathways synthesizing secondary metabolites, suggesting that this gene proximity is important to mycotoxin production.

**Figure 9:**
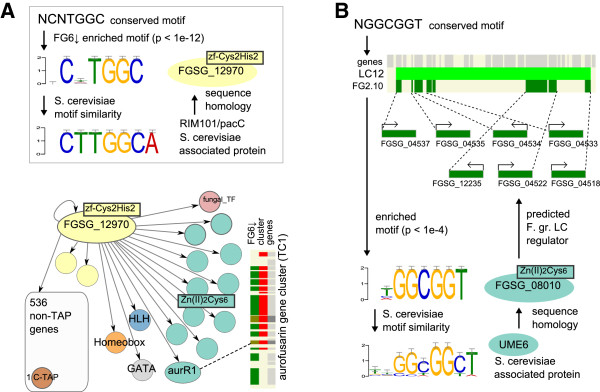
**Examples of potential transcriptional networks. (A)** A top-tier/’broad’-domain regulator (FGSG_12970) is shown with its putative binding site (sequence logo) in the promoters of genes comprising the FG6↓ co-expression group together with its similarity to the *S. cerevisiae* RIM101/PacC DNA-binding site. An arrow indicates possible interactions between the regulator and target promoters, with circles indicating second-tier regulators and their fill colours representing DBDs as described in Figure 8A. One second-tier regulator, *aurR1*, regulates the TC1 biosynthetic cluster (indicated by dotted line). **(B)** The sequence logo for a ‘narrow’-domain regulator (FGSG_08010) is displayed with its homologous UME6 binding site in budding yeast and putative target genes in the FG2.10 co-expression group and lying in the LC12 cluster.

Garber *et al.*[46] propose that in animals, transcription factors exhibit a multilayered architecture: Pioneer factors initiate remodelling of the epigenome, allowing broad binders to prime lineage-specific genes, with dynamic factors facilitating the activation of environment-specific genes. This layering - though in a much less complex and more compressed manner - is observed with the transcription networks/co-expression groups studied in this analysis; global regulators (mostly containing Cys_2_His_2_ and HLH DBDs) could play an analogous role to Pioneer and broad binders (Figure 9A), with factors predominately containing the Zn(II)_2_Cys_6_ DBD (only found in Fungi) activating small subsets of genes functioning in metabolism and development (Figure 9B).

## Methods

### Identification of *Fusarium graminearum* transcription-associated proteins

Genes encoding proteins associated with all aspects of the transcriptional process in *F. graminearum* were identified by querying the protein sequence entries of its genome [17] with two different types of data set [47,48]:

(i) A reference set of transcription–associated proteins (TAPs) was assembled from the UniProt database [49] by extracting entries whose GO terms are linked to transcription. The reference set sequences were filtered for compositional bias using CAST [50], and then used to search the *F. graminearum* genome with BLASTp [51]; any sequence similarities with an *E*-value ≤10^–6^ were considered as significant. To identify *F. graminearum* TAPs, the *Fusarium* homologues and their matching reference–set TAPs were clustered using TRIBE-MCL [52] at an inflation value of 2.0, as described previously [53] - this parameter value was chosen to minimize cluster granularity and ensure maximum coverage of the corresponding protein families. Any *F. graminearum* sequences present in the detected protein families (that also contain reference TAPs), were placed into one of five TAP categories based on the functional annotation of the reference sequences.

(ii) Profile hidden–Markov models (HMMs) of domains present in the proteins that constitute the Transfac database of well-characterised eukaryotic transcriptional factors [54]**,** in addition to those of DNA–binding domains present in all three domains of life [55], were used to search the *F. graminearum* genome (with HMMER [56]); any sequences that matched a HMM with a score greater than the lowest score for sequences included in the Pfam [57] full alignment of the family, were considered as hits. Based on the Pfam database records of the HMMs, these hits (TAPs) were placed into one of the five categories defined by the TAP reference set.

These complimentary sequence-comparison approaches identified a total of 723 TAPs in the *F. graminearum* genome (Additional file 1: Table S1).

### Detection of *F. graminearum* TAP orthologues

The 723 *F. graminearum* TAPs were used to query 56 eukaryotic genomes (BLASTp). Additionally, all the HMMs comprising the Pfam database were matched against the TAPs and these genomes. A sequence was considered to be orthologous to a *Fusarium* TAP if :-

(i) its protein-domain structure (defined by multiply-matching HMMs) is conserved entirely with a TAP, and ≥60% of this target sequence aligns with ≥60% of the query TAP,

(ii) ≥70% of it aligns to ≥70% of a TAP, both proteins match the same (single) HMM and neither match HMMs not present in both,

(iii) ≥80% of both TAP and query align and neither match an HMM.

### Microarray data analysis

Raw expression data (CEL files) from selected Affymetrix Fusariuma520094 GeneChip studies (FG1, FG2 [58], FG5 [59], FG6 [60]) were retrieved from PLEXdb [61]. These experiments were selected to represent a large part of the *Fusarium* life cycle. Each experiment was normalised independently to preclude batch effects which would obscure gene expression patterns [62]. Data sets FG2, FG5 and FG6 were quantile normalized using RMA (affy [63,64], R/Bioconductor [65]). To correct for increasing *Fusarium* hyphal biomass during the course of FG1 [58], a variance-stabilising model [66] was fitted to standardise the mean expression levels of RNA polymerase subunits. This procedure allows for both increases and decreases in gene expression to be detected using linear models of differential expression. Probeset detection calls were obtained using mas5calls [64,67]. Present, marginal and absent calls on replicate arrays were scored 1, 0.5, 0, respectively and called as detected if mean score across replicates > 0.6 [59]. Differential expression of probesets was determined using the limma package [68,69] with contrasts comparing each condition to the first time point or to complete media, using the minimum control probe *p*-value as the differential expression threshold [69].

### Genomic clustering of co-expressed genes

The *F. graminearum* coding sequences were mapped to chromosome position using BLAT and displayed using FgraMap (OmniMapFree [20]). Chromosomal clustering methods for co-expressed genes were based on gene order with a background gene list comprising the 13,773 genes represented on the Affymetrix microarray. Within each co-expression group (Figure 4) the distance (number of genes) from each member to the nearest member was stored; this generated a vector of pair-wise distances whose values were then compared with *g*_*prox*_, an integer parameter that was varied from 1 to 200 (Figure 5A). With each iteration of *g*_*prox*_, the number of elements in the vector whose value is less than or equal to *g*_*prox*_ was calculated (*N*_*gprox*_). The significance was evaluated using a *p*-value obtained from 1000 randomly sampled gene lists of the same size as the co-expression group. A *p*-value of less than 0.05 indicates that the observed *N*_*gprox*_ or greater was seen in fewer than 5% of randomly drawn gene lists, and a multiple testing correction was applied within each co-expression group [70]. For each co-expression group exhibiting a corrected significant value of *N*_*gprox*_ (*p* < 0.05) a *Z*-score was used to estimate conservatively how many of the observed proximal genes may be sufficient to explain the elevated value of *N*_*gprox*_. A threshold value of three standard deviations (*σ*) from the mean (*μ*) was obtained for the *N*_*gprox*_ null distribution (with *μ* and *σ* obtained from the randomly sampled gene lists). The difference *N*_*gprox*_*– (μ + 3σ)* provides an indication of the excess of proximal genes observed in the co-expression group compared with the resampled gene lists (Additional file 2: Table S3).

### TAP-centred (TC) clusters of co-expressed genes

For each differentially expressed TAP (Figure 4) the presence of neighbouring genes in the same co-expression group as the TAP was investigated using a variable window size of 2 to 40 genes, i.e. 1 to 20 genes on each side of the TAP (Figure 5B). For a given window size, Fisher’s exact test was used to determine the significance of enrichment for co-expression group members within the window. Within each co-expression group, *p*-values were corrected for multiple testing [71] and windows with *p* < 0.05 were considered significantly enriched. Where nested windows were significantly enriched, the largest such window size was identified for each TAP (Figures 5D and 6).

### Localized clusters (LC) of co-expressed genes

The Positional Gene Enrichment (PGE) tool was used to detect regions of the genome enriched for co-expression groups, with the emphasis on identifying localized clusters [30]. PGE was used to estimate a null distribution for each of the 22 co-expression groups by generating 10,000 random gene lists of equal size to the group: for each random list the most significant region and its associated enrichment *p*-value (min-*p*_*i*_) was returned. A *p*-value threshold was determined for each of the 22 min-*p*_*i*_ distributions at the 5%-ile of the 10,000 min-*p*_*i*_ values. PGE was then run on each co-expression group gene list, and regions were reported as significantly enriched localized clusters (LCs) only if the associated enrichment *p*-value was smaller than the corresponding threshold; nested or overlapping regions were merged (Figures 5D and 7).

### Functional annotation of genes present in TAP-centred and localized clusters

Putative functions for the proteins encoded by the 170 non-TAP genes present in the TCs and LCs were obtained by querying the 56 eukaryotic genomes, and clustering these sequences and their homologues as described above. The matches of the Pfam HMMs to the eukaryotic genomes enabled GO terms to be assigned to the TC and LC genes. Furthermore, querying the UniProtKB [49] with the gene identifiers of eukaryotic homologues present in the detected protein families allowed their functional annotations to be transferred to the *Fusarium* sequences. The clade specificity of each protein family was assigned as ‘Fusarium’, ‘Pezizomycotina’, ‘Fungi’, ‘non-metazoan Eukaryotes’ or ‘Eukaryotes’ using the NCBI taxonomic descriptions of these UniProtKB entries [72]. The predicted functional annotation for the TC/LC genes is provided in Additional file 1: Table S5.

### Enrichment of conserved upstream motifs and similarity to yeast motifs

Kumar *et al.*[33] reported 326 motifs located in upstream promoter regions of *F. graminearum* genes and conserved in *Fusarium* genomes, and summarized by motif similarity. Motif occurrence in upstream promoter regions (at least one forward or reverse-pair motif) was tested for enrichment amongst genes in each co-expression group, TC and LC. Following Kumar *et al.*[33], the upstream promoter region was defined as up to 600 bp upstream of each gene but excluding any overlapping upstream gene. 12,257 of the gene identifiers represented on the microarray were mapped uniquely to upstream promoter sequences (FG3 assembly [73]) and defined the universe of upstream regions for motif enrichments. The threshold for significant enrichment in co-expression groups was *p-*value < 10^-5^ (Fisher’s exact, one-tailed test) with an estimated false discovery rate of ~9% based on enrichment of permuted motifs (Additional file 2: Figure S3A). Upstream regions of genes in each TC and LC were tested for motif enrichment with significance threshold *p*-value < 10^-3^ (Additional file 2: Figure S3B). Tomtom (v4.8.1) [74] was used to detect similarity to yeast motifs (databases MacIsaac_v1 [75] and SCPD [76]; E < 1 and motif length ≤ 9) and to identify *S. cerevisiae* motif-associated proteins (ScAPs). *F. graminearum* homologues of ScAPs were identified (BLASTp; E < 10^-6^ and matched regions covering ≥ 30% of the query or target sequence).

## Abbreviations

DBD: DNA-binding domain; DON: Deoxynivalenol; D-TAP: DNA-binding transcription-associated protein; LC: Localized cluster; TAP: Transcription-associated protein; TC: TAP-centred Cluster; PGE: Positional Gene Enrichment; pTF: Phenotype-associated transcription factor.

## Competing interests

The authors declare that they have no competing interests.

## Authors’ contributions

KL performed the transcriptomics, gene cluster and statistical analyses, contributed to the study design and helped to draft the manuscript. KH-K contributed to the design of the gene cluster analysis. AB participated in the study design and helped to draft the manuscript. RMRC performed the TAP identification and annotation, designed the study and wrote the manuscript. All authors read and approved the final manuscript.

## Supplementary Material

Additional file 1 Contains Tables S1, S2, S5 and S6. Click here for file

Additional file 2 Contains Tables S3, S4 and S7, Supplementary Table legends, and Figures S1, S2 and S3. Click here for file
