# Supplementary material for: Genomic clustering and co-regulation of transcriptional networks in the pathogenic fungus Fusarium graminearum
Source: BMC Syst Biol. 2013 Jun 27;7:52. doi: 10.1186/1752-0509-7-52 (PMC3703260; doi:10.1186/1752-0509-7-52)

Supplementary Materials

Lawler et al.

Supplementary Table Legends

Supplementary Table 1. [Supplementary file .XLS] Transcription-associated proteins in the *F. graminearum* genome. Columns are the gene identifier (FG1) with hyperlink to MIPS FGDB ('geneID.MIPS'), gene identifier (FG3) with hyperlink to EnsemblFungi ('geneID.EnsemblFungi'), TAP class (B, C, D, O, P), TRIBE-MCL cluster identifier, HMM protein family with link to Pfam, and functional annotation transferred from homologous genes where there is no HMM protein family indicated. See SupplementaryFile2.xls (SupplTable1_TAPs).

Supplementary Table 2. [Supplementary file .XLS] Gene members of each co-expression group. Columns are the gene identifier (FG1) with hyperlink to MIPS FGDB ('geneID.MIPS'), gene identifier (FG3) with hyperlink to EnsemblFungi ('geneID.EnsemblFungi'), TAP class and HMM protein family for transcription-associated proteins, co-expression group membership ('expt.FG1', … ,'expt.FG6') and associated probesets. Probesets mapping to more than one gene were discarded and are not shown. Co-expression group key (see Fig. 4): 'stays_up' = ↑; 'stays_down' = ↓; 'transient_up' = ↑↓; 'transient_down' = ↓↑. See SupplementaryFile2.xls (SupplTable2_coexprGroups).

Supplementary Table 3. Chromosomal clustering of co-expression groups within a background of all annotated *F. graminearum* genes.

Supplementary Table 4. Summary of TAP-centred clusters (TCs) and localized clusters (LCs).

Supplementary Table 5. [Supplementary file .XLS] Functional annotation of co-expressed members of TCs and LCs. Columns are the FG1 gene identifier, TRIBE-MCL cluster identifier, taxonomic specificity, TRIBE-MCL cluster size and breakdown by taxonomic specificity of each TRIBE-MCL cluster member, annotation and associated GO terms. See SupplementaryFile2.xls (SupplTable5_AnnotationTCsLCs).

Supplementary Table 6. [Supplementary file .XLS] Motifs enriched in one or more co-expression groups with respect to the FG3 genome build ('Universe'). Columns show co-expression group

(‘coexprGrp’), motif (‘motif’), motif cluster (as in Kumar *et al.*), number of genes in the co-expression group for which the motif occurs in the promotor region (‘counts_univ_coexprGrp’), size of the co-expression group, (‘coexprGrp_sizeInUniverse’), number of genes in the genome for which the motif occurs in the promotor region (‘counts_univ’), odds ratio and p-value for enrichment (Fisher’s exact test). See SupplementaryFile2.xls (SupplTable6_motifEnrich)

Supplementary Table 7. Conserved *F. graminearum* DNA-motifs enriched in a co-expression group and associated with a putative transcriptional regulator.

Supplementary Figure Legends

Supplementary Figure 1. Chromosomal locations of the members of nine co-expression groups which exhibit significant clustering on the F. graminearum genome.

Supplementary Figure 2. Overview of methods to find enriched conserved *F. graminearum* DNA-motifs and associated putative global regulators.

Supplementary Figure 3. Significance thresholds for motif enrichment in a gene list.

Supplementary Table 3. Lawler et al.

Supplementary Table 3. Chromosomal clustering of co-expression groups within a background of all annotated F. graminearum genes. Of the 22 co-expression groups (Fig. 4), nine showed evidence of non-random clustering when considering the proximity of each gene member to the nearest member (see Fig. 5A and Methods). The observed number of genes lying within gprox genes of a co-expressed gene was compared to 1000 randomly drawn gene lists, resulting in an empirical null distribution. For each of the nine co-expression groups showing significant clustering ($p<0.05$), the p-value and Z-score are reported, together with the observed number of genes with proximity *gprox* = 1 .. 10 (obs) and the minimum number of such genes required for significance at Z = 3 (mean + 3 std. dev. where mean and std. dev. are from the empirical null distribution) ($exp_{Z=3}$). For example, in FG2.01, 52 gene members lie adjacent to a co-expressed gene (*gprox* = 1), with empirical p-value 0. An observed value of 34 adjecent co-expressed genes would achieve a Z-score of 3.

**Co-expression group**	**proximity:**	*gprox* = 1	2	3	4	5	6	7	8	9	10
FG1↑	p-value (Z-score)		0 (5.80)	0 (4.55)	0 (4.60)	0 (3.62)	1e-04 (3.55)	1e-04 (4.34)	2e-04 (3.77)		
	obs ($exp_{Z=3}$)		245 (203)	299 (274)	362 (335)	398 (388)	443 (434)	495 (474)	521 (509)		
FG2.01	p-value (Z-score)	0 (6.17)	0 (5.68)	0 (4.81)	1e-04 (4.12)						
	obs ($exp_{Z=3}$)	52 (34)	76 (56)	91 (75)	103 (92)						
FG2.10	p-value (Z-score)	0 (10.40)	0 (8.40)	0 (7.28)	0 (5.60)	0 (4.94)	0 (4.75)	0 (4.38)	0 (4.46)		
	obs ($exp_{Z=3}$)	112 (56)	148 (94)	175 (127)	188 (156)	207 (182)	229 (206)	246 (228)	267 (248)		
FG2.-1-1	p-value (Z-score)	0 (5.10)	0 (5.02)	1e-04 (4.27)	0 (4.23)	2e-04 (3.69)					
	obs ($exp_{Z=3}$)	23 (16)	35 (26)	41 (34)	49 (41)	53 (48)					
FG2.11	p-value (Z-score)	0 (7.07)	1e-04 (5.02)	0 (4.94)							
	obs ($exp_{Z=3}$)	25 (13)	29 (21)	37 (28)							
FG5↓	p-value (Z-score)	0 (11.00)	0 (8.24)	0 (6.34)	0 (5.36)	2e-04 (3.81)	0 (4.39)	0 (4.44)	0 (4.55)	0 (4.37)	4e-04 (3.67)
	obs ($exp_{Z=3}$)	101 (46)	123 (76)	138 (104)	154 (128)	159 (149)	186 (169)	205 (187)	223 (204)	236 (219)	242 (234)
FG5↑	p-value (Z-score)	0 (6.40)	0 (6.24)	0 (4.41)	0 (3.86)						
	obs ($exp_{Z=3}$)	170 (128)	268 (218)	317 (294)	373 (358)						
FG5↑↓	p-value (Z-score)	1e-04 (4.24)	0 (4.48)	0 (5.30)	0 (4.51)	0 (4.42)	0 (4.20)	0 (4.10)	0 (4.03)	1e-04 (3.75)	
	obs ($exp_{Z=3}$)	36 (30)	58 (48)	83 (65)	93 (80)	107 (93)	118 (106)	129 (118)	139 (128)	146 (138)	
FG6↓	p-value (Z-score)	0 (10.39)	0 (8.47)	0 (7.99)	0 (6.64)	0 (5.82)	0 (4.89)	0 (5.36)	0 (4.55)	0 (4.47)	1e-04 (3.88)
	obs ($exp_{Z=3}$)	209 (121)	286 (205)	357 (277)	399 (339)	439 (392)	470 (439)	517 (480)	539 (516)	568 (547)	587 (575)

Supplementary Table 4. Summary of TAP-centred clusters (TCs) and localized clusters (LCs). Each detected cluster is summarized as the number of FgraMap genes within each cluster span (see Figs. 6, 7), the number and percentage of FgraMap genes which are co-expressed, and the breakdown of co-expressed genes by taxonomic specificity (see Methods). Fus, Fusarium-specific; Pez, Pezizomycotina; Fungi + nonME, Fungi + non-metazoan Eukaryotes; Euk, Eukaryotes.

Detected cluster	# FgraMap genes	# co-expr genes	% co-expr genes	Fus	%Fus	Pez	%Pez	Fungi	%Fungi	Fungi + nonME	% Fungi + nonME	Euk	%Euk
TC1	26	14	54%	2	14%	2	14%	2	14%		0%	8	57%
TC2	4	4	100%	1	25%	1	25%		0%		0%	2	50%
TC3	24	11	46%	4	36%	1	9%	3	27%		0%	3	27%
TC4	5	5	100%	4	80%		0%		0%		0%	1	20%
TC5	3	3	100%		0%		0%	2	67%		0%	1	33%
TC6	5	4	80%		0%		0%	2	50%		0%	2	50%
TC7	4	4	100%	4	100%		0%		0%		0%		0%
LC1	24	11	46%	4	36%	1	9%	3	27%		0%	3	27%
LC2LC3	96	20	21%	7	35%	1	5%	3	15%		0%	9	45%
LC4	24	10	42%	7	70%		0%	2	20%		0%	1	10%
LC5	44	15	34%	3	20%	3	20%	2	13%	2	13%	5	33%
LC6	55	10	18%	1	10%	3	30%	1	10%		0%	5	50%
LC7	6	6	100%	1	17%	5	83%		0%		0%		0%
LC8	75	17	23%	1	6%	1	6%	2	12%		0%	13	76%
LC9	98	22	22%	3	14%	9	41%	5	23%		0%	5	23%
LC10	63	16	25%	3	19%	4	25%	3	19%		0%	6	38%
LC11	4	4	100%		0%	3	75%		0%	1	25%		0%
LC12	21	8	38%	5	63%	2	25%	1	13%		0%		0%
LC13	7	5	71%	2	40%		0%	1	20%		0%	2	40%
LC14	2	2	100%		0%	1	50%		0%	1	50%		0%
LC15	5	5	100%		0%		0%	2	40%	1	20%	2	40%
LC16	138	12	9%	2	17%	1	8%	2	17%	2	17%	5	42%
LC17	3	3	100%		0%		0%	2	67%	1	33%		0%

Supplementary Table 7, Lawler et al.

Supplementary Table 7. Conserved *F. graminearum* DNA-motifs (Kumar *et al.* 2010 enriched in a co-expression group and associated with a putative transcriptional regulator. A putative regulator was assigned to an enriched motif if a homologous transcription factor in *S. cerevisiae* is associated with a similar DNA-motif (see Supplementary Figure 2 for overview). The table shows the enriched *F. graminearum* motif and motif cluster (as in Kumar *et al.* 2010), the enriched co-expression groups (Supplementary Table 6), the associated putative *F. graminearum* regulator(s) and *S. cerevisiae* homologue(s) (in brackets), and the associated *F. graminearum* DNA-binding domain ('DBD').

**Motif**	**Motif cluster**	**#Co-expr groups**	**Co-expr group**	**Fgr identifier (Scer name)**	**DBD**
CCCCTSN	M1	1	FG1↑	FGSG_06871 (MSN4)	zf-C2H2
GTGGGGN	M1	1	FG1↑	FGSG_01350 (MET31)	zf-C2H2
CCNCCNC	M1	1	FG1↑	FGSG_02743 (ADR1)	zf-C2H2
GNGGGGY	M1	2	FG2.-10<br>FG2.-11	FGSG_06871 (MSN2)<br>FGSG_06871 (MSN2)	zf-C2H2<br>zf-C2H2
NNCCCCT	M1	1	FG6↓	FGSG_06871 (MSN4)	zf-C2H2
CACGTGN	M2	1	FG1↑	FGSG_02814 (TYE7)/FGSG_05567 (CBF1) /FGSG_00750 (INO4)	HLH
CAYGTGA	M2	1	FG2.11	FGSG_02814 (TYE7)/FGSG_05567 (CBF1) /FGSG_00750 (INO4)/FGSG_09043 (MATALPHA2)	HLH/Homeobox/zf-C2H2
CGTCAYN	M3	1	FG1↑	FGSG_01307 (RTG3)	HLH
NTSACGT	M3	1	FG1↑	FGSG_02814 (TYE7)/FGSG_05567 (CBF1)	HLH
ACGTCAY	M3	3	FG6↓↑<br>FG6↓	FGSG_02814 (TYE7)/FGSG_05567 (CBF1)<br>FGSG_05567 (CBF1)	HLH<br>HLH
NCGTCAY	M3	1	FG6↓↑	FGSG_01307 (RTG3)	HLH
ACGTCAN	M3	1	FG6↓	FGSG_02814 (TYE7)	HLH
CGCGNCN	M4		FG2.11	FGSG_04220 (SWI6)/FGSG_10384 (MBP1)	APSES
CNGCCNC	M5	1	FG1↑	FGSG_01341 (CRZ1)	zf-C2H2
NCCGCCN	M5	1	FG1↑	FGSG_08010 (UME6)	Zn_clus
CGCCANN	M5	1	FG2.10	FGSG_08010 (UME6)	Zn_clus
NGTGGCK	M5	1	FG2.10	FGSG_01341 (CRZ1)	zf-C2H2
NTGYGGC	M5	2	FG2.10<br>FG5↑↓	FGSG_01350 (MET31)<br>FGSG_01341 (CRZ1)	zf-C2H2<br>zf-C2H2
CNGGGGN	M6	1	FG1↑	FGSG_06871 (MSN2)	zf-C2H2
CCCCNGN	M6	1	FG6↓	FGSG_06871 (MSN2)	zf-C2H2
GCNNGGC	M12	2	FG1↑<br>FG6↓	FGSG_06359 (SKN7)<br>FGSG_12970 (RIM101)	HSF_DNA-bind<br>zf_C2H2
ACGCSNC	M13	1	FG2.11	FGSG_04220 (SWI6)/FGSG_10384 (MBP1)	APSES
CCSGCCN	M14	1	FG1↑	FGSG_08010 (UME6)	Zn_clus
NACCCCG	M17	1	FG2.01	FGSG_02743 (ADR1)/FGSG_13911 (REB1)	zf-C2H2/Myb_DNA-binding
NGNGGCT	M23	1	FG2.10	FGSG_08010 (UME6)/FGSG_01341 (CRZ1)	Zn_clus/zf-C2H2
YGATAAG	M26	1	FG2.01	FGSG_08634 (DAL80)	GATA
NCNTGGC	M38	1	FG6↓	FGSG_06359 (SKN7)/FGSG_12970 (RIM101)	HSF_DNA-bind/zf_C2H2
NTCAGCC	M41	1	FG6↓	FGSG_01341 (CRZ1)	zf-C2H2
GGYTGGN	M58	1	FG6↓	FGSG_06311 (ACE2)	zf-C2H2
AGGGKYC	M61	1	FG6↓	FGSG_01298 (NRG1)	zf-C2H2
NAGGGNT	M61	1	FG6↓	FGSG_06871 (MSN2)	zf-C2H2

Motif	Motif cluster	#TCs/LCs	TCs/LCs	Fgr identifier (Scer name)	DNA-binding domain
NATRACG	M3	1	LC10	FGSG_01307 (RTG3)	HLH
CGGNCCG	M18	1	TC1/LC5	FGSG_02531 (STB4)	Zn_clus
NGGCGGT	M34	1	LC12	FGSG_08010 (UME6)	Zn_clus
CNTGGCR	M38	1	LC10	FGSG_12970 (RIM101)	zf-C2H2
TGACTMA	M53	1	LC6	FGSG_08696 (ARG80)/FGSG_03214 (ARG81) /FGSG_09286 (GCN4)/FGSG_01307 (RTG3)	SRF-TF/Zn_clus /bZIP/HLH

Supplementary Figure 1. Lawler et al.

Supplementary Figure 1. Chromosomal locations of the members of nine co-expression groups which exhibit significant clustering on the *F. graminearum* genome. Recombination rates and gene density are also shown.

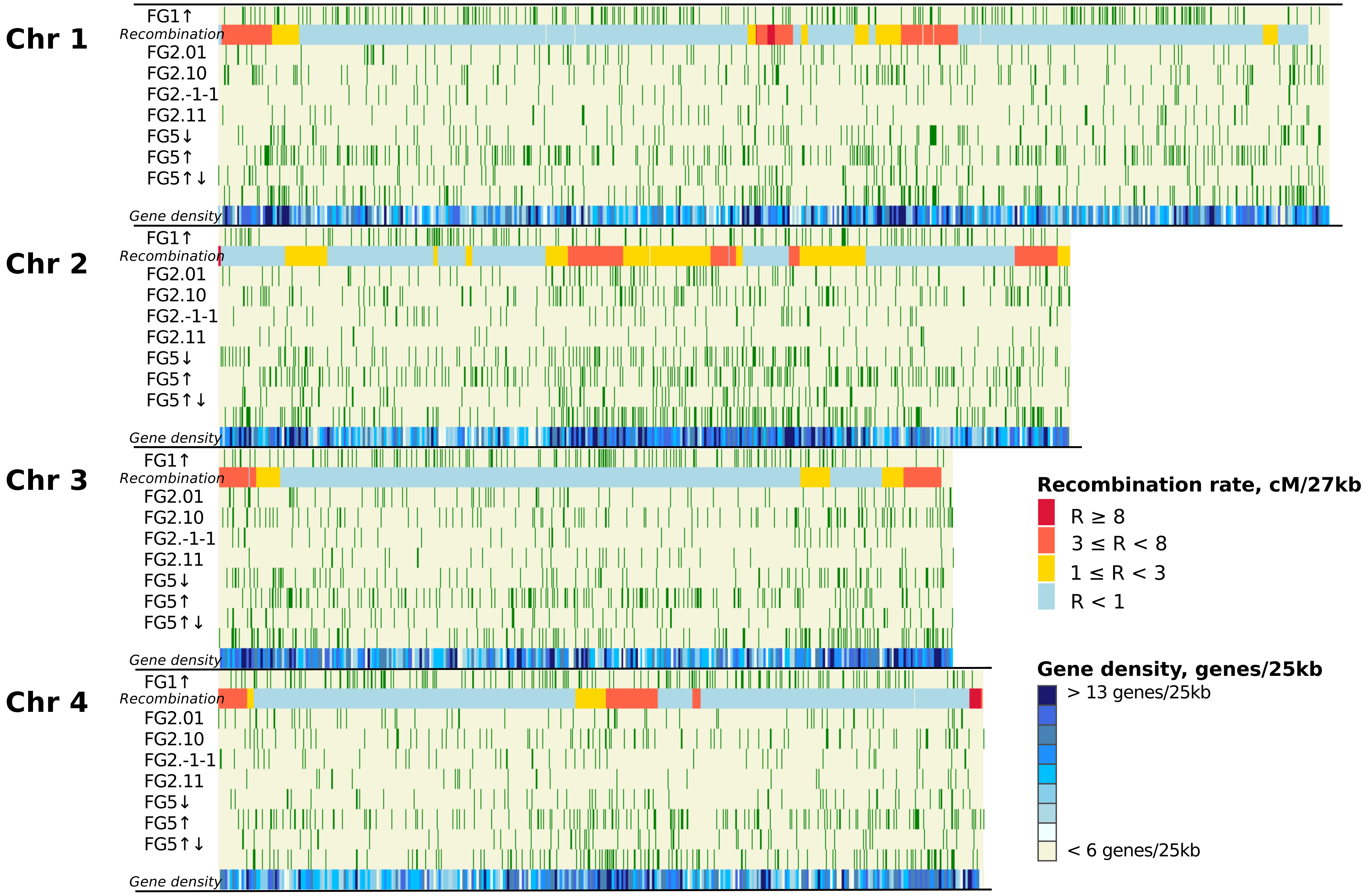

Supplementary Figure 2, Lawler et al.

Supplementary Figure 2. Overview of methods to find enriched conserved *F. graminearum* DNA-motifs and associated putative global regulators.

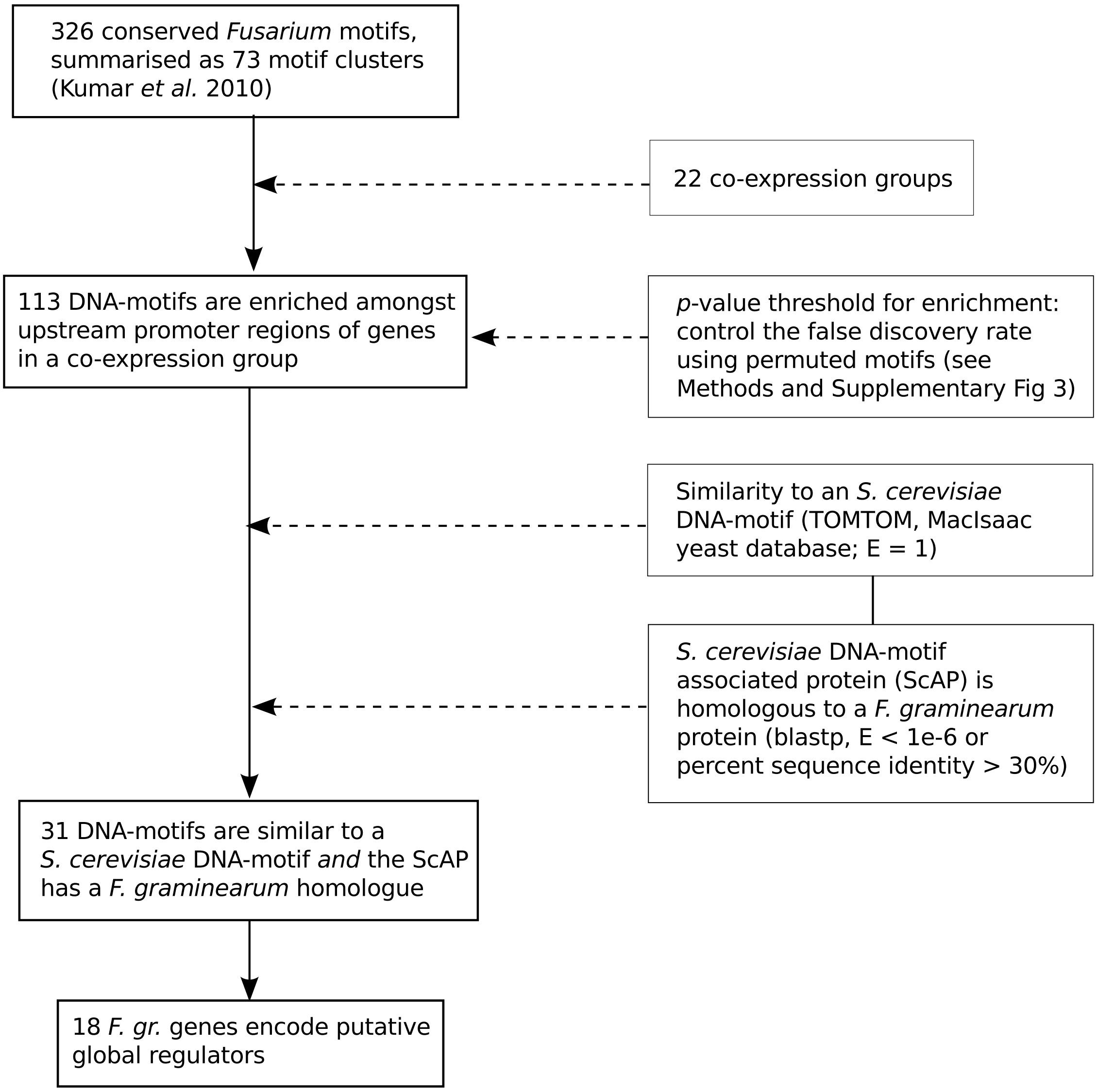

Supplementary Figure 3. Lawler et al.

Supplementary Figure 2. Significance thresholds for motif enrichment in a gene list. A. The number of times a motif is enriched in a coexpression group (all degenerate motifs, Kumar et al. 2010 [1]) compared with the number of times a permuted motif is enriched in a coexpression group. Permuted motifs were obtained by permuting each motif character [A, C, G, T] -> [C, T, A, G] or the equivalent permutation for two-fold degenerate characters [1]. At enrichment p-value=1e-5 (dashed vertical line), a motif was enriched in a coexpression group 156 times compared with 13 times amongst permuted motifs, suggesting a false discovery rate of ~9%. By comparison, enrichment p-values 1e-6 and 1e-4 lead to false discovery rate estimates of ~5% (100 motif enrichments, 5 permuted motif enrichment) and 16% (230 motif enrichments compared with 35 permuted motif enrichments) respectively. B. TCs and LCs contain fewer genes than the co-expression groups, reducing the power to achieve genome-wide significance. A threshold of 1e-3 was taken as the significance threshold for motif enrichment.

A

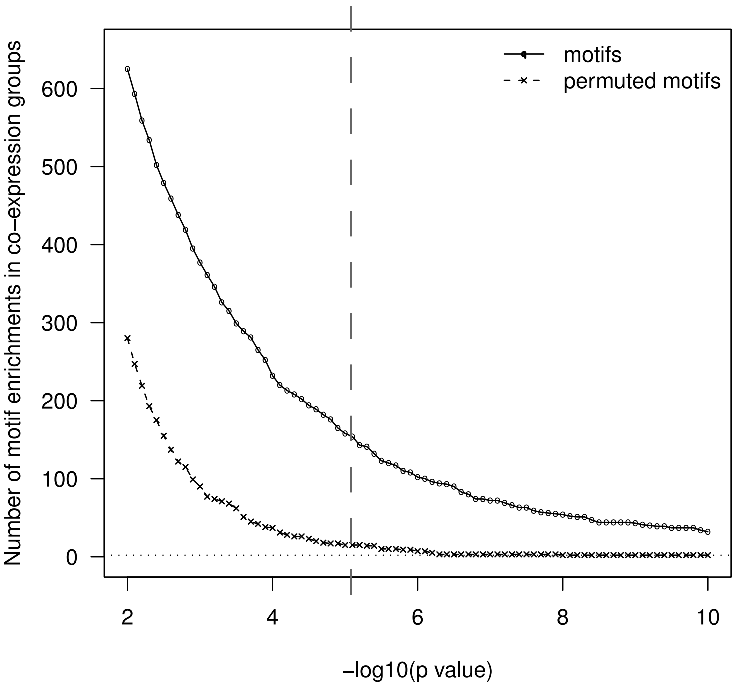

B

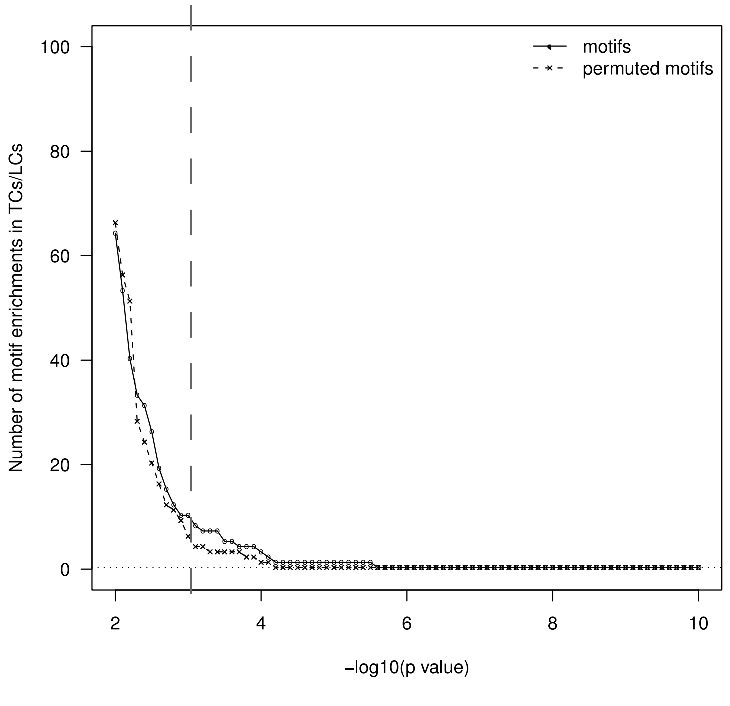

Supplement: Additional file 2 — Contains Tables S3, S4 and S7, Supplementary Table legends, and Figures S1, S2 and S3. [file 1752-0509-7-52-S2.pdf]
